# Editorial: Advances in the potential treatments of gastrointestinal and liver diseases: addressing the public health burden

**DOI:** 10.3389/fphar.2023.1253069

**Published:** 2023-07-13

**Authors:** Adina Turcu-Stiolica, Maria Dimitrova, Mariana Jinga

**Affiliations:** ^1^ Department of Pharmacoeconomics, University of Medicine and Pharmacy of Craiova, Craiova, Romania; ^2^ Department of Organization and Economy of Pharmacy, Medical University Sofia, Sofia, Bulgaria; ^3^ Department of Gastroenterology, Carol Davila University of Medicine and Pharmacy, Bucharest, Romania

**Keywords:** gastrointestinal, machine learning, plants, efficacy, molecules

Gastrointestinal and liver diseases continue to pose a significant burden worldwide, both on societies and healthcare systems (Marcellin and Kutala, 2018). With recent advances in studying the pathogenesis of these diseases and the development of therapy and screening strategies for early detection, physicians, pharmacists and policymakers can now address this major public health burden. This includes not only for cancer but also chronic liver diseases and autoimmune diseases (Goebel et al., 2015; World Health Organization, 2022). By doing so, they can improve the therapeutics outcomes, enhance the quality of life of patients, and optimize healthcare costs.

The present Research Topic consists of twenty articles: 3 systematic reviews with meta-analysis, 3 reviews, and 14 original research articles. These articles provide a diverse range of innovative viewpoints on the significance of comprehending the genetic, molecular, and cellular mechanisms associated with gastrointestinal complications and diseases. Such understanding has the potential to guide the advancement of therapies in areas where clinical problems remain unsolved.

Regarding cellular and molecular mechanisms, Sui et al. examined the relationship between various immune cells and postoperative ileus, aiming to provide potential therapeutic solutions for this unresolved clinical issue. Jiang et al. analyzed the potential antifibrotic effects of salvianolic acid B in autophagy in liver fibrosis. Their findings revealed that inducing TGF-β1 resulted in a significant increase in autophagosome formation and autophagic flux in different molecular cascades. Another original research studied by Jia et al. demonstrated the *in vivo* efficacy of hesperidin in promoting gastric motility in rats with functional dyspepsia, suggesting a potential treatment approach for this condition.

Screening and early detection are among the primary priorities of the current European anticancer plan and should be implemented at the national level (Pana et al., 2023). Within our Research Topic, three original research articles emphasized the significance of on national level screening. Groza et al. conducted a study analysing the quality of colonoscopy in Romania as part of colorectal cancer screening. They discovered that the quality of colonoscopy is not consistently monitored and suggested using adenoma detection rate, polyp detection rate, and adenoma per colonoscopy as monitoring indicators. In their systematic review with meta-analysis, Ding et al. examined the regimes that could improve bowel cleansing quality for patients undergoing colonoscopy. They emphasized the importance of adequately preparing patients before screening interventions to improve the quality of the results. Additionally, Fu et al. provided a review describing the current diagnostic methods, therapeutic targets, and therapies for non-alcoholic fatty liver disease.

Irritable bowel syndrome (IBS) is a functional gastrointestinal disorder. Bao et al. demonstrated that genetic susceptibility to insomnia could increase the risk of IBS. However, they did not observe a causal association between chronotype and IBS, nor did they find that genetic liability to the “morning” chronotype could lower the risk of IBS.

Many of the original research articles contributing to this Research Topic also provided insights on the potential pharmacological effects of plant-derived medicines. Li et al. conducted a review on the current progress of plant-derived treatments such as terpenoids, flavonoids, quinones, *etc.*, against intestinal mucositis. He et al. analyzed traditional Chinese medicines as a potential source of hepatoprotective agents. An interesting aspect of their original research was the use of an *in silico* model based on machine learning techniques to discover potential pharmacologically active plants. Afolabi et al. examined methanolic Moringa oleifera leaf extract for its potential protective role against epithelial barrier damage and enteric bacterial translocation in intestinal ischaemia/reperfusion injury. Chen et al. demonstrated that the patchouli alcohol, extracted from *Pogostemonis Herba*, improves intestinal motility and alleviates IBS-induced diarrheal symptoms. These findings suggest the potential therapeutic efficacy of patchouli alcohol against IBS accompanied with diarrhea.

Network pharmacology and molecular docking are novel methods that contribute to a comprehensive understanding of systems and network biology, as well as the orientation of molecules within binding sites. Currently, these methods are primarily used in the discovery of plant-based, marine-based, and antibiotics drugs (Aleem et al., 2022). In our Research Topic, Xia et al. have contributed with a research article that employs these methods to explore the potential *in vivo* protective effect of Epidemii Folium on cisplatin-induced intestinal injury in mice. Their findings showed that the extract of Epidemii Folium can alleviate cisplatin-induced intestinal damage by modulating oxidative stress, inflammation, and apoptotis.

The utilisation of artificial intelligence and machine-learning in gastroenterology allows the creation of models for predicting prognostics such as mortality (Ungureanu et al., 2023) or liver protection. He et al. employed these techniques to predict the hepatoprotective activity of ingredients derived from 12 traditional Chinese medicine, which could aid in the development of new drugs.

Two original research articles examined the efficacy and safety as major criteria for evidence-based therapy decision making. Li et al. investigated the efficacy of mesalazine in treating nonspecific terminal ileal ulcers and demonstrated no significant difference in clinical or endoscopic efficacy between patients who received mesalazine and those who were followed up without special intervention. Sur et al. analyzed the efficacy and safety of trifluridine/tipiracil as a therapeutic option in metastatic colorectal cancer in real life setting for a Romanian cohort of patients. Liu et al. conducted a meta-analysis showing that neoadjuvant imatinib plus surgery significantly improve overall survival of rectal gastrointestinal stromal tumor compared to upfront surgery. Another meta-analysis, performed by Zhang et al., focused on changes in hemodynamic and cardiac function in patients with portal pulmonary hypertension (POPH) to understand the effect of pulmonary hypertension agents (including prostacyclin and its analogues, endothelin receptor antagonists, phosphodiesterase 5 inhibitors, soluble guanylate cyclase stimulants, *etc.*) treatment on the entire population of POPH patients.

Primary biliary cholangitis (PBC), a chronic autoimmune intrahepatic cholestatic disease, is still not fully understood. Meta-analyses were performed to better understand its pathogenic mechanisms (Ionele et al., 2022). Guoyun et al. updated a meta-analysis to explore the influence of fenofibrate dose and the effectiveness and safety of long-term application on PBC patients, with the aim of improving guideline recommendations.

In the field of gastrointestinal endoscopy, Zheng et al. determined the optimal dose of propofol combined with esketamine for gastroscopy in elderly patients.

In summary, the articles presented in this Research Topic focus on molecular and cellular mechanisms, the efficacy and safety of established therapies in real-world settings and provide insight into potential therapies derived from plant origins, as well as advanced solutions for screening, diagnosis, and treatment of gastrointestinal and liver diseases.
